# Rare Complication of Interventional Radiology-guided Arterial Embolization of the Gastroduodenal Artery in the Setting of Acute Gastrointestinal Bleed: Migrated Coils in the Duodenum

**DOI:** 10.7759/cureus.7365

**Published:** 2020-03-22

**Authors:** Pujitha Kudaravalli, Sheikh A Saleem, Venkata Satish Pendela, Muhammad Osman Arif

**Affiliations:** 1 Internal Medicine, State University of New York (SUNY) Upstate Medical University, Syracuse, USA; 2 Gastroenterology, State University of New York (SUNY) Upstate Medical University, Syracuse, USA; 3 Internal Medicine, Rochester General Hospital, Rochester, USA

**Keywords:** migrated coils, complication, infection, interventional radiology guided embolization

## Abstract

A 91-year-old male presented to the emergency room with hemodynamically significant upper gastrointestinal bleeding. The patient underwent an esophagogastroduodenoscopy (EGD), which showed frank blood in the duodenum interfering with the visualization. Hence, the patient underwent urgent interventional radiology (IR)-guided arteriogram and embolization. An EGD done 48 hours later showed a giant, non-bleeding, cratered duodenal ulcer with a visible vessel and vascular coils partially protruding into the duodenal bulb lumen. The patient had no evidence of bleeding post embolization. The patient presented three months later with abdominal pain. Computed tomography (CT) abdomen showed multiple liver abscesses. IR-guided drainage of abscesses was performed, and the culture grew Streptococcus intermedius. Magnetic resonance cholangiopancreatography (MRCP), endoscopic retrograde cholangiopancreatography (ERCP), and barium enema were unremarkable. The patient was treated with a prolonged course of intravenous (IV) antibiotics and recovered without any further issues. IR guided arterial embolization can be lifesaving in cases where GI bleeding cannot be controlled endoscopically, however, it can lead to serious complications, including endovascular coil migration into the gastrointestinal (GI) lumen causing infection and re-bleeding. Endovascular coil migration can occur immediately or several years later, which can result in fatal bleeding and infection. The best approach to prevent and manage migrated endovascular coils in the GI lumen remains unclear.

## Introduction

Acute gastrointestinal (GI) bleeding continues to be a significant economic health burden, with an incidence rate of 90-108 per 100,000 persons and a mortality rate of 3%-14% in the United States [1]. Interventional radiology (IR)-guided embolotherapy can be lifesaving when an acute GI bleed cannot be controlled with medical or endoscopic therapy. However, IR embolization can lead to various complications and, herein, we present a rare complication.

## Case presentation

A 91-year-old male with a past medical history of coronary artery disease, benign prostatic hyperplasia, hypertension, and history of cholecystectomy and depression was transferred to the emergency room from another hospital with significant hematemesis and melena. The patient was taken to the outside hospital with the above-mentioned symptoms when he was administered two units of packed red blood cells (PRBCs) for hemoglobin of 6.8 g/dL and hematocrit of 21.3%.

The patient was hemodynamically stable on arrival with a blood pressure of 120/70 mm Hg and a heart rate of 83 beats/minute. A few hours after admission, the patient had two more episodes of hematemesis without a palpable pulse and CPR was initiated. The patient regained pulse shortly after the initiation of cardiopulmonary resuscitation (CPR). A repeat laboratory work-up showed a hemoglobin of 6.6 g/dL with a hematocrit of 18%, blood urea nitrogen (BUN) of 46 mg/dL, and creatinine of 0.99 mg/dL. The patient was administered two more units of PRBCs and was started on pressor support for the stabilization of blood pressure.

**Figure 1:**
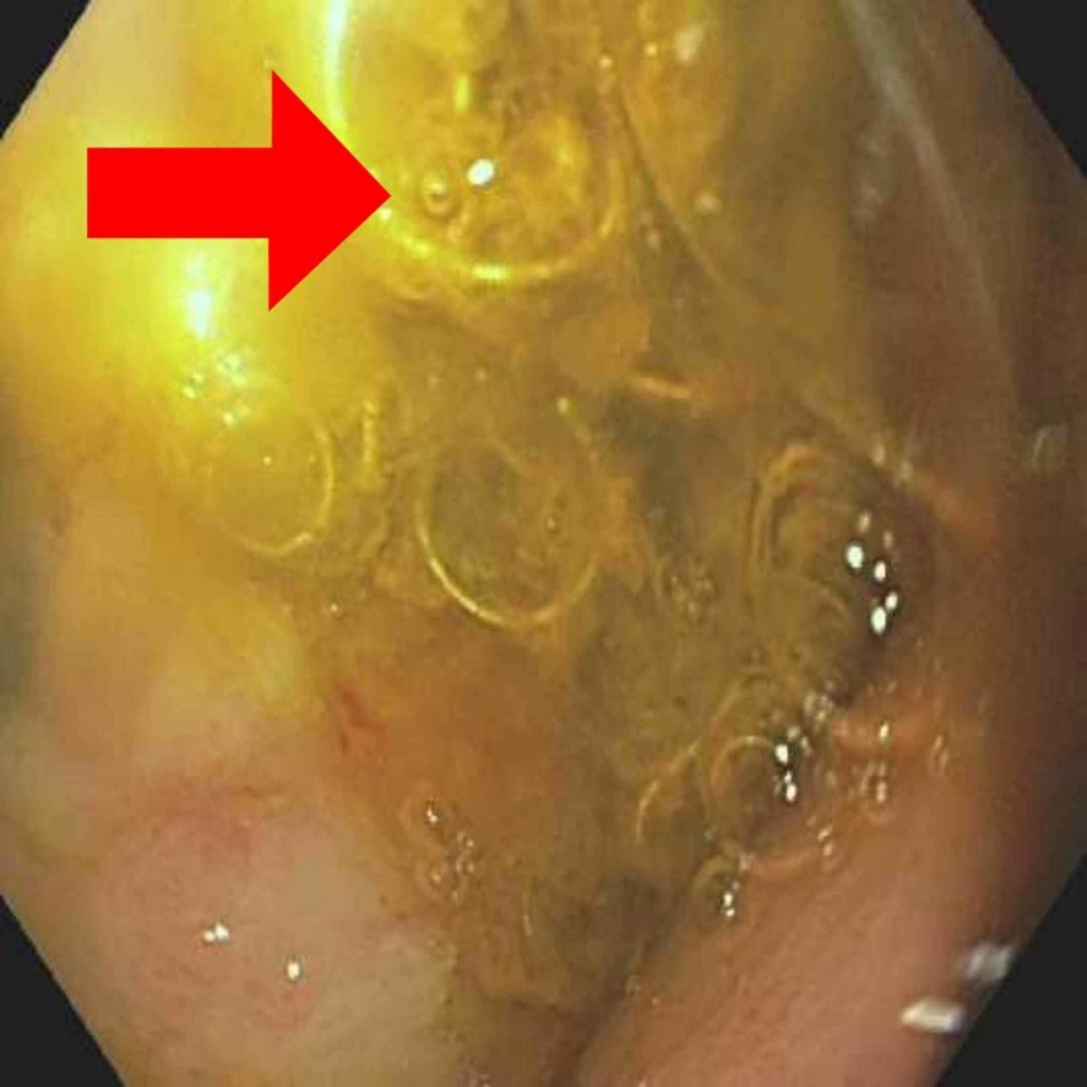
Vascular coil Endoscopy showing protrusion of vascular coils at the duodenal bulb

After medical optimization of the patient, he underwent upper GI endoscopy (EGD), which showed a large amount of frank blood in the duodenum interfering with the visualization and treatment of the bleeding site. Hence, the patient underwent an urgent IR-guided arteriogram and embolization of the gastroduodenal artery with the placement of vascular coils. An EGD was done 48 hours later, which showed a giant non-bleeding cratered duodenal ulcer with a visible vessel and vascular coils partially protruding into the duodenal bulb lumen (Figure 1). No active bleeding was noted from the ulcer. The patient had no further evidence of bleeding post embolization and was discharged [2].

**Figure 2:**
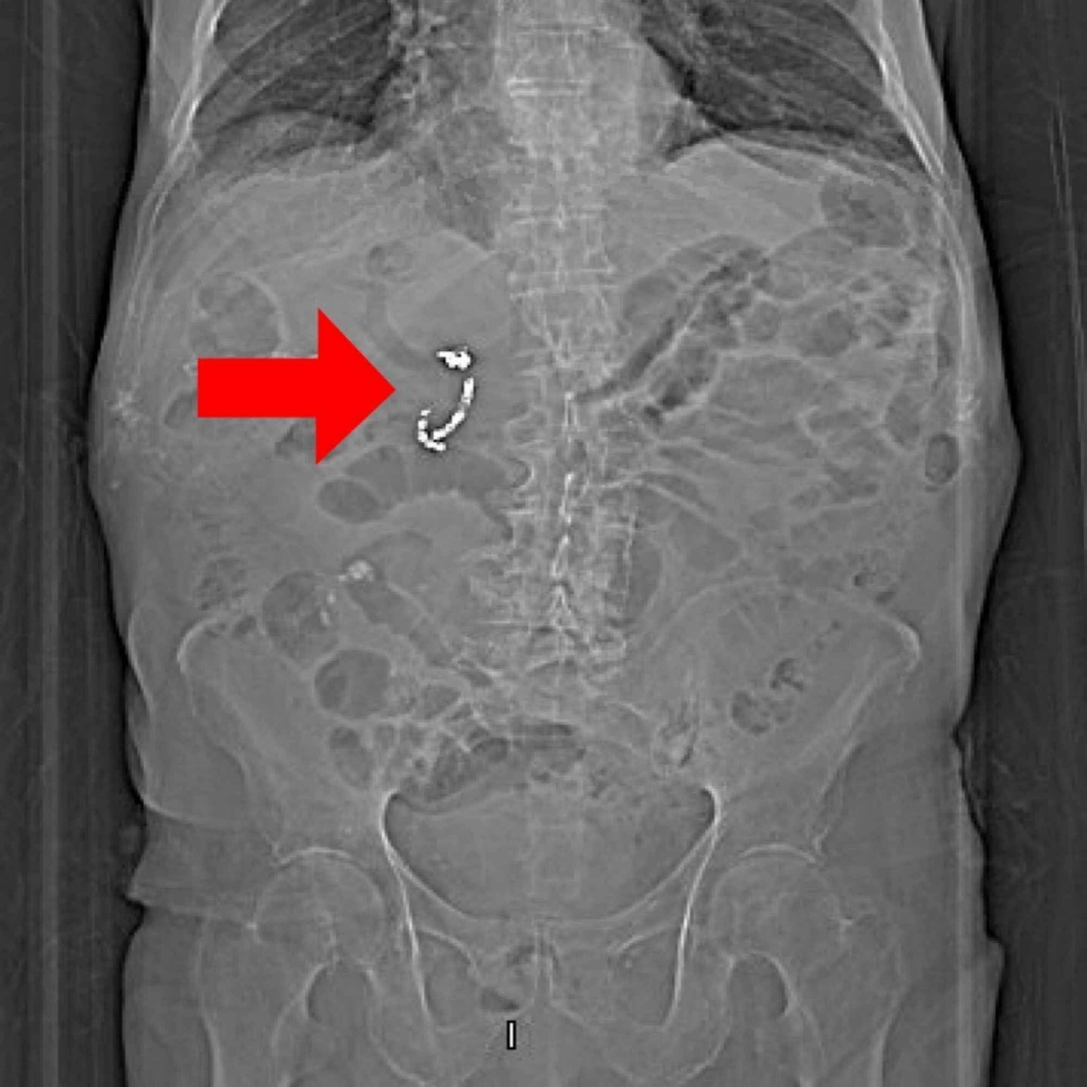
Vascular coil: radiolographical image Abdominal computed tomography showing a vascular coil

The patient presented three months later with right upper quadrant abdominal pain. Abdominal tenderness was present at the right upper quadrant on palpation. White blood cell (WBC) count was elevated at 19.9 with a left shift, hemoglobin was 9.1 g/dL, hematocrit was 28.4%, lipase was 20 U/L, aspartate aminotransferase (AST) was 88 U/L, ALT was 38 U/L, alkaline phosphatase (ALP) was 75 U/L, total bilirubin was 0.6 mg/dL. Computed tomography (CT) of the abdomen was obtained, which showed a large 8 cm x 12 cm collection of fluid along the anterior capsule of the left hepatic lobe and a loculated collection posterior to the right upper transverse colon. It also showed the vascular coils (Figure 2). IR-guided drainage of abscesses was performed, and the culture grew Streptococcus intermedius. Magnetic resonance cholangiopancreatography (MRCP) and endoscopic retrograde cholangiopancreatography (ERCP) were obtained to evaluate for biliary obstruction, and they were unremarkable. Barium enema was sought to evaluate for a possible colonic source of infection, and it was negative as well. The patient was treated with a prolonged course of intravenous (IV) antibiotics and recovered without any further issues.

## Discussion

IR-guided arterial embolization can be lifesaving in cases where GI bleeding cannot be controlled with medical or endoscopic intervention. It is both diagnostic and therapeutic and is shown to have lower 30-day mortality in comparison to surgery [3]. The common femoral artery is used to gain access for endovascular angiography. Computed tomographic angiography and nuclear scintigraphy can aid the radiologist to locate the bleed. The celiac artery and its branches are the first vessels to be evaluated for bleeding, as gastroduodenal ulcers are the most common source of bleeding. If it is negative, the superior mesenteric artery is examined if the suspected source of bleeding is the proximal colon and the inferior mesenteric artery is examined if the source of bleeding is the distal colon. If all of the above are negative, the internal iliac arteries is examined to evaluate the middle and inferior rectal arteries, as they can be a source of bleeding. Extravasation of contrast is considered a sign of active bleeding [4]. Metallic coils, hydrogel particles, gel foam, acrylic glue, or a combination of these materials are used for embolization.

Complications of transarterial embolization (TAE) include access-site hematomas (3%-17%), pseudo-aneurysms, arterial dissection, ischemia (2.7%), and coil migration (3%). Although coil migration is rare, it is still a well-known complication. Coil migration is more likely to occur in cases of a pseudoaneurysm, aneurysm, and arterio-enteric fistulas. It is hypothesized that the ischemia of the bowel wall caused by the embolization of the artery allows the partial or complete migration of the coil into the bowel lumen. This could lead to fatal hemorrhage, bowel ischemia, or small bowel obstruction from the migration of a large number of coils. There are reports of coils migrating into the biliary tract causing biliary obstruction and acting as a potential nidus for infection [5-7].

The technique and type of material used influences the migration of the coil. In a pseudoaneurysm, occluding the normal portion of the artery proximally and distally instead of filling the pseudoaneurysm itself with coils has shown to decrease the incidence of migration. The use of the sandwich technique while deploying coils is shown to prevent coil migration [8].

The approach to the management of migrated coils has not been studied. Endovascular coil migration can occur immediately or several years later. Long-term follow-up with plain radiographs has been done to detect migration of coils in a few case reports [9]. Hot biopsy forceps was used to cut the wires and the migrated coils were successfully removed in one case report and another described using endoscopic scissors to cut the coils and extracting it in a piecemeal fashion with the help of raw-toothed forceps [8-10]. Bleeding is a major risk for the removal of migrated coils using the above procedures. The coils can also spontaneously pass through the rectum and conservative management is also recommended bearing in mind the risk of fatal re-bleeding. The best approach to prevent and manage migrated endovascular coils in the GI lumen remains unclear.

Our patient presented with abdominal pain three months after the initial procedure. The migration of gut bacteria through the ischemic bowel wall and mucosal abrasions caused by the migrated coils led to the development of liver abscesses. After treatment of the infection, it was decided to follow the patient conservatively due to the increased risk of bleeding when a migrated coil is removed [2].

## Conclusions

Migrated vascular coils is a rare but well-known complication. There are no treatment guidelines for the management of migrated coils and more research to formulate a treatment algorithm is required. Clinicians should bear in mind this rare complication.
